# Initiating levodopa pump therapy: a qualitative study of patient and caregiver perspectives

**DOI:** 10.1186/s12912-026-05527-6

**Published:** 2026-10-20

**Authors:** Monica Scharfenort, Anette Nobrell, Michela Garon, Kristina Rosqvist, Gesine Paul, Maria H. Nilsson, Angelo Antonini, Dag Nyholm, Per Odin, Elisabeth Mangrio

**Affiliations:** 1 Department of Clinical Sciences Lund, Faculty of Medicine, Lund University, Lund, Sweden; 2 Department of Neurology, Rehabilitation Medicine, Memory and Geriatrics, Skåne University Hospital, Lund, Sweden; 3 Neurodegenerative Disease Unit, Center for Rare Neurological Diseases (ERN- RND), Department of Neurosciences, Padua Neuroscience Center (PNC), University of Padova, Padova, Italy; 4 IRCCS San Camillo, Venice, Italy; 5 Department of Health Sciences, Faculty of Medicine, Lund University, Lund, Sweden; 6 Memory Clinic, Skåne University Hospital, Malmö, Sweden; 7 Department of Medical Sciences, Neurology, Uppsala University, Uppsala, Sweden; 8 Department of Care Sciences, Malmö University, Malmö, Sweden

**Keywords:** Parkinson’s disease, Pump therapy, Infusion, Patients, Family caregivers, Satisfaction, Expectations, Lived experiences, Qualitative research

## Abstract

**Background:**

Parkinson’s disease (PD) is a progressive neurodegenerative disorder affecting motor and non-motor functions. When oral medication no longer provides adequate control, device-aided therapies (DAT), including levodopa infusion therapy, may be introduced. Although DAT can improve symptom management, little is known about how people with PD (PwPD) and family caregivers (FC) experience the initiation process. This study explored PwPD and their FC perceptions during decision-making, therapy initiation, and subsequent adaptation to levodopa pump therapy.

**Methods:**

This qualitative study employed semi-structured interviews (6–24 months after treatment initiation) and latent reflexive thematic analysis with ten PwPD and nine FC recruited from two Swedish university hospitals.

**Results:**

Thematic analysis revealed four shared themes: (1) Living with the constraints of PD, (2) Sustaining hope in the face of therapeutic uncertainty, (3) Adapting to a new treatment while facing practical challenges, and (4) Relying on expertise while managing its absence. Pump therapy was often chosen due to limited alternatives and strong trust in the specialized multidisciplinary PD team. While both groups experienced symptom improvements, PwPD focused on autonomy whereas FC focused on responsibilities. Expectations were generally met, yet FC frequently felt underinformed about the realities of daily pump management.

**Conclusions:**

Our findings highlight that although symptom control improved, both PwPD and FC continued to face practical and emotional challenges. Supporting their adaptation process requires coordinated, person-centred preparation, guidance and follow-up from multidisciplinary, specialized PD teams.

**Clinical trial registration:**

Not applicable.

**Supplementary Information:**

The online version contains supplementary material available at https://doi.org/10.1186/s12912-026-05527-6.

## Background

Parkinson’s disease (PD) is a progressive neurodegenerative disorder characterized by motor symptoms: bradykinesia, rigidity, tremor, and postural instability; and non-motor symptoms such as cognitive decline, fatigue, and depression [1, 2]. As PD progresses, the effectiveness of oral levodopa therapy shortens, and daily functioning becomes increasingly impaired. To manage the increasingly short duration of effect, many patients are prescribed more frequent dosing. After about five years, up to 85% of individuals have more than four daily intake [3]. When symptom control is no longer adequate, device-aided therapies (DAT) may be introduced. Deep brain stimulation (DBS), continuous subcutaneous apomorphine infusion (CSAI), levodopa–carbidopa intestinal gel infusion with or without entacapone (LCIG/LECIG), and subcutaneous infusion of foslevodopa/foscarbidopa (LDp/CDp) have all demonstrated efficacy for motor and non-motor symptoms [4, 5]. In Sweden, national guidelines recommend that decisions regarding advanced therapies for PD are made within a multidisciplinary treatment conference. This approach ensures that professionals with expertise in different DAT options contribute to individualized, person-centred care [6]. Studies suggest that DAT may improve PD-related quality of life (QoL), although the magnitude of improvement varies across therapies and individuals [7]. However, some PwPD experience adverse effects and other treatment-related challenges after therapy initiation [8]. These experiences suggest that clinical outcomes alone do not fully capture treatment success. Beyond efficacy, satisfaction is influenced by: psychological factors (e.g. depression and self-efficacy) [9, 10], social and economic context [11], care processes (e.g., expertise and support) [12, 13] and demographic aspects such as age and education [14]. Coping strategies also affect well-being, although they tend to remain relatively stable over time [15].

Family caregivers (FC) often play a key role in treatment decisions and daily management. Their experiences both influence and are influenced by treatment outcomes. Caregiver burden typically increases with disease severity and disability [16]. Although DAT may reduce strain compared to oral regimens, the impact varies considerably [17–21]. Because decisions and adjustments occur within a care relationship, both patient and caregiver perspectives must inform person‑centred care [22, 23]. Despite these insights, little is known about how PwPD and FCs experience the initiation of levodopa-based pump therapy or how treatment adaptation is negotiated within the patient-caregiver relationship. The limited qualitative evidence suggests that users perceived the pump as reliable and associated it with improved daily control and reduced treatment burden. However, tubing issues, device weight, and practical management remained challenging [24–26]. However, these studies focused exclusively on PwPD and did not include FC perspectives or the relational dynamics that shape treatment adaptation. By bringing these perspectives together, the study aims to provide a more comprehensive understanding of how treatment adaptation, expectations, responsibilities and support needs are experienced and negotiated within an ongoing care relationship. The study also seeks to identify factors relevant to person-centred implementation and support. Throughout this article, the term “pump therapy” is used to refer specifically to levodopa-based pump therapies.

## Methods

The study has three objectives:

1. To explore how PwPD and their FC experience the process of initiating pump therapy.
2. To explore how they perceive the extent to which their expectations were fulfilled by the pump therapy.
3. To explore how they experience the ongoing adaptation to pump therapy.

### Design

This qualitative study used an inductive approach and semi-structured interviews, analysed via latent reflexive thematic analysis inspired by Braun and Clarke [27], to explore experiences related to initiating levodopa-based pump therapy in PD. Reporting followed the Consolidated Criteria for Reporting Qualitative Research (COREQ) guidelines [28].

### Participants & procedures

Participants were recruited as part of a retrospective follow-up to a previous, unpublished prospective study (ethical approval no. 2022-01412-01) conducted at Uppsala University Hospital and Skåne University Hospital in Lund, Sweden. The original cohort consisted of PwPD who, following routine multidisciplinary assessment for DAT, were offered and accepted levodopa-based pump therapy at the participating centres. The multidisciplinary assessment included evaluation of motor symptoms, non-motor symptoms, cognition, treatment response and motor fluctuations by a team comprising movement disorder specialists, PD nurses, physiotherapists, occupational therapists and neuropsychologists. Eligible participants for the present qualitative follow-up study were PwPD from this original cohort who had initiated pump therapy 6–24 months earlier. FC were identified by participating PwPD as relatives or close family members involved in ongoing care and support. In both studies, participating PwPD were asked to identify a family caregiver willing to participate. FC were defined as unpaid relatives or close family members providing ongoing care, including assistance with daily activities, symptom management, and coordination of healthcare, consistent with standard definitions of informal caregiving [22]. No specific cognitive inclusion or exclusion criteria were applied; however, all PwPD had undergone the routine multidisciplinary assessment described above. Of the 15 PwPD and 15 FC invited, 11 (5 PwPD, 6 FC) declined participation due to health issues, time constraints or lack of interest. In total, nineteen participants (10 PwPD and 9 FC) consented and completed the study. The participating FC consisted of eight spouses/partners and one adult child.

### Data collection

Individual semi-structured interviews were conducted between October 2024 and January 2025, guided by an interview guide developed by the research team (see Additional file 1). The interview guide was developed based on previous literature on experiences of device-aided therapies, clinical expertise within the multidisciplinary research team, and insights gained from the unpublished prospective study that formed the basis for the present follow-up study. The guide was further refined through a pilot interview with a recently initiated LDp/CDp user. This pilot interview was excluded from analysis of the current study due to differences in treatment modality and care routines. To describe participant characteristics, sociodemographic and clinical data were retrieved from the earlier prospective study and were not collected specifically for the present qualitative follow-up study. Clinical variables (including LEDD and OFF-time) were collected as part of the original prospective study before pump initiation and after 6 months of pump therapy. Variables included age, sex, marital status, years of formal education, disease duration, duration of pump therapy, daily medication dose and OFF-time assessed using MDS-UPDRS Part IV, Item 4.3.

The first author (MS, female, PhD student and experienced PD nurse trained in qualitative methods) interviewed all PwPD. The second author (AN, female, MSc, similarly trained) interviewed all FC. Interviews were conducted at participants’ preferred location, either in their homes or at the clinic. PwPD interviews lasted on average 39 min (min-max 35–43) and FC interviews averaged 34 min (min-max 26–53). All interviews were audio-recorded and transcribed verbatim. No other persons were present during the interviews, and no repeated interviews were conducted.

### Data analysis

The analytic process followed Braun and Clarke’s six steps: (1) *familiarization with the data*; through repeated transcript reading; (2) *generating initial codes*; by identifying condensed meaning units (3) *searching for themes*; by grouping related codes (4) *reviewing themes*; and refining themes continuously (5) *defining and naming* overarching themes, and (6) *producing the report* with interpretative engagement. An example illustrating the link between a meaning unit, its code and the corresponding subtheme/theme is provided in Table 1. In this example, the code was grouped with other codes describing medication burden and the demands of frequent symptom management, forming the subtheme “Pills, pills, pills!”, which contributed to the broader theme “Living with the constraints of PD”. The analysis involved iterative movement between coded extracts, subthemes, and the developing thematic structure. Codes expressing similar underlying meanings were clustered into preliminary subthemes, which were subsequently reviewed and compared across transcripts. Themes were developed through interpretive engagement with the dataset and refined to capture patterns of shared meaning across participants rather than individual interview topics. MS and AN conducted the initial coding and theme development. They first coded the transcripts independently and subsequently compared and discussed their interpretations. MG (PhD student and neuropsychologist with qualitative analysis experience) contributed to coding discussions and refinement of subthemes and themes. Regular analytic meetings were held to review coded extracts, discuss alternative interpretations, and refine theme boundaries. To enhance credibility, an external qualitative researcher (EM), not involved in data collection and with limited neurological expertise, reviewed the coding structure, theme development, and supporting quotations and challenged emerging interpretations. Final themes were developed and refined through iterative discussions within the research team.

Trustworthiness was guided by Lincoln and Guba’s criteria for credibility, confirmability and transferability [29]. Reflexive discussions, researcher triangulation, and iterative comparisons between codes, themes, and original transcripts were used throughout the analytic process. Transcripts were not returned to participants, no participant checking was performed, no repeated interviews were conducted and analysis was carried out manually without qualitative software.

Descriptive statistics were used to summarise participant characteristics. Continuous variables are presented as median and interquartile range (IQR; q1–q3), and categorical variables as counts and percentages.

All interviews were conducted and transcribed in Swedish. The initial coding and thematic analysis were performed on the Swedish transcripts by MS and AN. To enable collaborative coding discussions and theme refinement, the full transcripts were translated into English by MS and AN. This provided MG with complete contextual access to the data when contributing to coding and theme development. The external qualitative expert (EM) had access to both the Swedish transcripts and the English translations and reviewed the analytic process and translated material to enhance credibility.

**Table 1:** Illustration of how a meaning unit develops into a theme in a family caregiver interview

Meaning caring unit	Condensed unit	Code	Subtheme	Theme
*“No*,* but because it was*,* you know*,* a tablet every 90 minutes. And that*,* and I mean*,* it’s not easy to remember*,* right? No*,* because it was a constant thinking about time*,* I would say. Because I also knew that if he forgot about it—because*,* my God*,* it’s not easy for [PwPD] to remember either—no*,* and… and I mean*,* if [PwPD] forgot about it*,* you know*,* then [PwPD] would feel bad*,* yeah.”*	*Remembering to take medication every 90 min was difficult*,* both for the PwPD and the FC*,* as missed doses led to worsening symptoms.*	*Daily life organised around medication routines*	*Pills*,* pills*,* pills!*	*Living with the constraints of PD*

### Ethical considerations

The study was approved by the Swedish Ethical Review Authority (2024-03848-02) and conducted in accordance with the Declaration of Helsinki. Written informed consent was obtained prior to data collection and participants were informed that they could withdraw from the study at any time without providing a reason. Data were pseudonymized using participant codes (e.g., P01–P10), and identifying details were replaced with generic terms.

## Results

Participant characteristics are summarized in Table 2.

**Table 2:** Participant characteristics

	PwPD			FC
**Total**	10			9
**Sex** (female/male), n (% male))	5/5 (50)			4/5 (56)
**Age** (years), Median (q1-q3)	72.5 (61–78)			75 (64.5–78)
**Years of formal education**, Median (q1-q3)	12 (10.8–14.3)			13 (12.5–16)
**Relationship with PwPD**				
Spouse/Partner, n (%)				8 (89)
Child, n (%)				1 (11)
**Disease duration** (years), Median (q1-q3)	12 (10.8–14.3)			
**Duration of pump therapy** (months), Median (q1-q3)	16.5 (11.5–20.25)			
**Treatment regime** 16 h out of 24, n (%) 24 h out of 24, n (%)	8 (80) 2 (20)			
**LEDD** (mg/day) Before pump, Median (q1 – q3) 6 months with pump, Median (q1 – q3)	1112.9 (954.0–1347.6) 1467.2 (1029.6–1589.0)			
**Time in OFF** (MDS-UPDRS Part IV score, Item 4.3), Median (q1 – q3) Before pump 6 months with pump	2 (1.8–3) 1 (0.8–2)			

Note. PwPD People with Parkinson’s Disease, FC family caregiver, Pump therapy Levodopa/Carbidopa Intestinal gel with or without Entacapone, LEDD Levodopa Equivalent Daily Dose (including all antiparkinsonian medication regardless of levodopa-based pump formulation), MDS-UPDRS Movement Disorders Society – Unified Parkinson’s Disease Rating Scale. Item 4.3 (Part IV): Time spent in the OFF state (scored from 0–4 (higher= worse); 0 = none, 4 = ≥ 76%)

Thematic analysis resulted in four overarching themes shared by both PwPD and FC: (1) *Living with the constraints of PD* (2) *Sustaining hope in the face of therapeutic uncertainty*, (3) *Adapting to a new treatment while facing practical challenges*, (4) *Relying on expertise while managing its absence*. These are summarized with subthemes in Table 3 (for illustrative quotes, see Additional file 2).

**Table 3:** Summary of themes and subthemes from both people with Parkinson’s and their family caregivers

Main theme	PwPD subtheme	FC Subtheme
Living with the constraints of PD	Symptoms of PD	Emotional implications
	The cycle of loss	Shrinking world
	Pills, pills, pills!	Pills, pills, pills!
Sustaining hope in the face of therapeutic uncertainty	Information & communication about DAT	Information & communication about DAT
	Expectations about pump	Expectations about pump
	Feeling cornered	Feeling cornered
	Satisfaction of pump	Satisfaction of pump
Adapting to a new treatment while facing practical challenges	Starting with Naso	Inserting Naso & PEG
	Effect of the pump	Effect of the pump
	Adjusting with pump	Adjusting with pump
	Residual and Emerging Symptoms	Residual and Emerging Symptoms
	Impact on social relations	
Relying on expertise while managing its absence	HCP (lack of) knowledge about pump	HCP (lack of) knowledge about the pump
	Support from PD/pump support nurses	Support from PD/pump support nurses
		FC support: giving and receiving

Note. PD Parkinson’s Disease, PwPD People with PD, FC family caregivers, DAT Device-aided therapy, Naso Nasojejunal tube, PEG Percutaneous Endoscopic Gastrostomy, HCP healthcare professionals

### Living with the constraints of Parkinson’s disease

This theme captures how progressive symptom burden, increasing medication demands, and reduced spontaneity constrained everyday life for both PwPD and FC. Although these experiences were expressed differently by PwPD and FC, they reflected a shared experience of adapting to a life increasingly shaped by PD. Participants described how PD disrupted daily life for both PwPD and FC prior to pump initiation, although the emotional and practical implications differed. This was reflected in accounts of motor fluctuations, increasing medication demands, and reduced spontaneity, all of which constrained social participation and everyday autonomy.

Before, I think, it was more like a rollercoaster, with peaks and then you roll straight – bang, crash! Then you have to climb up again. That’s just how it is when you rely only on [oral] medication. (PwPD9)

FC often assumed responsibility for medication timing, adding to the practical burden of care.

Over time, the effect of oral medication became shorter-lasting, leading to more frequent wearing-off periods for PwPD. This imposed rigid scheduling and created stress for both PwPD and FC as even minor delays in dosing could cause rapid re‑emergence of motor and non‑motor symptoms. Managing medication timing became a central concern for both PwPD and FC, with late or missed doses potentially leading to rapid symptom deterioration.

You had to keep track of [PwPD] medications. You had to be like: Have you taken a tablet now? Do you remember now? Yeah, and you know, when you’re in the middle of doing other things, caught up in something—well, then you lose track of it…. Yeah, and then everything turns to crap immediately. (FC4)

Recurring themes included loss of independence, reduced spontaneity and withdrawal from both social and physical activities, affecting both parties. For FC, the unpredictability created emotional strain and a sense of a shrinking personal world. Many described being trapped in a cycle of anticipation and reaction to symptoms:

No, no, not at all. You couldn’t, you can’t do anything, you don’t get in the car, you can’t go anywhere, you can’t visit friends and acquaintances. (FC5)

Although pump therapy later improved certain symptoms and introduced new possibilities, such as short trips or moments of spontaneity, FC noted that habits of caution often lingered.

### Sustaining hope in the face of therapeutic uncertainty

This theme describes how decisions about pump therapy were shaped by a hope for improvement, trust in healthcare professionals, and a perceived lack of alternative treatment options. While both PwPD and FC hoped to achieve better symptom control, their expectations differed somewhat. PwPD primarily emphasized symptom relief and regained autonomy, whereas FC balanced hope for improvement with concerns about new caregiving responsibilities.

Initiation of pump therapy was typically guided by the PD team at the university hospital. Only a few participants sought peer input; most relied primarily on the team’s expertise, even when they viewed the decision as personal or shared with family. Despite strong trust in clinicians, uncertainty about treatment outcomes remained, fostering a cautiously optimistic stance toward pump therapy:

They said, like, well, you won’t be cured of your Parkinson’s just because you get the pump. So, I didn’t have such high expectations, but still, I did. I thought like this: no tablets! That’s what I thought. (FC8)

After initiation, symptom control was generally improved. Some, however, were disappointed by delayed therapeutic effects and limited practical preparation. Once treatment began, many noted a marked improvement in motor symptoms:

Like I said, I jumped into bed and walked without my walker. It was like, ‘Yes! Let’s do this!’(PwPD 7)

But now, everything is good [PwPD1] is already walking without a walker and comes to meet you in the hallway. And I’m so happy. (FC1)

Despite overall satisfaction, many felt they had received insufficient information about the practical demands of pump therapy, their responsibilities in managing the device, and the lengthy adjustment period before optimal effects emerged. Although participants understood that the pump would not offer a complete solution, some still hoped for a more pronounced therapeutic benefit:

Yes, exactly, it’s really difficult to get a clear idea of how and what it actually involves. It was more about the appearance. And we have to admit that it’s one thing to look at the equipment, but it’s a whole other thing to actually handle it. (FC5)

Although they acknowledged several difficulties, all participants reported that they would recommend pump therapy, each for different reasons. PwPD cited improved mobility and reduced pill burden; FC valued stability and regained personal time.

Go for it. I should have said it right away, don’t hesitate for a second, not even a second. Just go for it, if you get the chance. No doubt about it. No. (FC4)

### Adapting to a new treatment while facing practical challenges

This theme reflects participants’ experiences of learning to live with pump therapy, balancing symptom improvements against practical challenges, side effects, and the ongoing process of treatment adjustment. While the pump often improved stability and daily functioning, participants also described a demanding initiation period and the need to adapt to residual symptoms and new practical responsibilities. Initiation of LCIG/LECIG involved a nasojejunal tube (Naso) trial before offering Percutaneous Endoscopic Gastrostomy (PEG) placement (including jejunal extension tube) for intestinal infusion. Naso initiation was often distressing; some PwPD developed sores, and one nearly declined therapy due to discomfort.

PwPD8: The most unpleasant part of all of it [starting pump therapy] was probably the tube in my nose.

Interviewer: That was the worst part?

PwPD8: Yeah, hell yeah! That was awful!

PEG placement also introduced challenges, including bleeding requiring reinsertion and instances in which side effects were initially misattributed. Therapeutic effects ranged from clearly beneficial to uncertain, and most participants required a period of dose optimization. In one case, severe side effects, including hallucinations, necessitated hospitalization, illustrating that dose optimization was not always straightforward. Symptoms persisted or emerged over time and were often most pronounced at night and in the morning, before pump activation, or after physical exertion, often prompting supplemental oral medication or dose adjustments.

We take those sustained-release tablets in the evening before going to bed. And they work for at least half the night, but then it becomes difficult. So, that’s a problem. [PwPD] has to wear a diaper, otherwise, it won’t work. (FC7)

Many PwPD regained the ability to act more spontaneously, yet daily activities often still required planning, particularly when medication had to be transported and stored safely. The type of residual symptom also mattered. When memory disturbances or hallucinations occurred, participants described them as closely resembling the pre-treatment phase. Some acknowledged that personal fears restricted their engagement more than the treatment itself. In addition to these symptom-related challenges, many FC described the practical demands of managing the pump. Seven of the nine FC assumed responsibility for tasks such as flushing the tube system, cleaning the PEG stoma, and monitoring supplies, and reported difficulties including leakage, staining, odours, and poor equipment design. These routines added complexity to daily life, and management materials were often criticized for poor ergonomics, visibility, and comfort, leading some participants to view the system as incomplete or insufficiently integrated:

Okay, now I’ve made a pump. It’s the other stuff that’s important! How am I supposed to refine it further [referring to company management and FC desire for their strategic thinking]? And then there’s that damn little triangle [triangular retention plate]. (FC9)

In response, several developed their own adaptations to facilitate daily care.

### Relying on expertise while managing its absence

This theme captures how access to knowledgeable healthcare professionals influences adaptation to pump therapy and how participants often assume responsibility when expertise is unavailable. Experiences varied considerably depending on whether participants encountered specialist PD services or healthcare professionals with limited knowledge of pump therapy. While PD and pump support nurses were described as important sources of expertise and reassurance, insufficient knowledge among other healthcare professionals often shifted responsibility to PwPD and FC.

Participants valued the specialized support provided by PD- and pump nurses during initiation, but this level of access diminished over time. As specialist availability decreased, some relied on instruction manuals to manage technical issues. This often left both PwPD and FC feeling increasingly responsible and isolated, especially when complications arose or when general HCPs were unfamiliar with the device. One PwPD described a situation where a nurse attempted to flush the PEG incorrectly and had to call for guidance:

The staff… they had no…had very little knowledge. And one evening, it was the first evening I had had it [the PEG] inserted, a nurse was supposed to help me flush it. She had never done it before. She had to call someone to ask how to do it. And she put it in the wrong port [gastric port]. (PwPD10)

For FC, the treatment was not only about learning a new medication routine but also about mastering technical aspects of the pump system. This dual responsibility, handling medication effects and troubleshooting equipment failures such as leakage or pump malfunctions, created a strong need for ongoing support. As one FC explained:

Yes, but it’s that, it’s not just the medication, there’s a bit of mechanics in the crap as well. (FC5)

This quote illustrates the unexpected technical burden that accompanied the therapies. Limited knowledge among general HCPs sometimes resulted in delays and additional stress for FC, who occasionally felt responsible for guiding healthcare professionals through technical procedures.

## Discussion

This study highlights three main findings. First, adaptation to levodopa-based pump therapy was shaped not only by symptom improvement but also by practical and emotional challenges experienced by both PwPD and FC. Second, expectations and satisfaction were closely linked to trust in specialist healthcare teams and perceptions of limited treatment alternatives. Third, successful adaptation depended heavily on access to ongoing specialist support, while limited knowledge among non-specialist healthcare professionals often shifted responsibility to patients and caregivers. By incorporating both PwPD and FC perspectives, this study extends existing knowledge of adaptation to advanced therapies by highlighting how treatment experiences are negotiated within an ongoing care relationship.

### Finding 1: Adaptation extends beyond symptom control

Before pump initiation, participants described lives increasingly constrained by symptom fluctuations, medication schedules, and reduced spontaneity. Consistent with previous literature, these challenges affected both PwPD and FC, although in different ways: PwPD primarily emphasized loss of autonomy while FC described increasing responsibility and emotional burden [16, 21, 30–32]. Our findings extend this literature by showing how these challenges were experienced within an ongoing care relationship, where symptom management increasingly shifted responsibility from PwPD to FC and shaped expectations for advanced therapies. Previous studies have highlighted the importance of treatment goals and shared decision-making in DAT selection [23, 33]. Similarly, participants in our study hoped for improved symptom control, but decisions were also influenced by organizational factors and a perceived lack of alternatives, creating a sense of inevitability rather than choice for some individuals [25, 32, 34]. Notably, they reported receiving information about all DAT options, which contrasts with previous real‑world findings where shared decision‑making has been restricted by incomplete information [23]. Given that participants had lived with PD for a median of 12 years and had used pump therapy for a median of 16.5 months, their accounts provide insight into adaptation to therapy beyond the immediate initiation phase.

The initiation phase was described as physically and emotionally demanding, particularly during the Naso trial. Discomfort and complications such as sore throats or reflux led some to consider discontinuing therapy, mirroring reports of the burden associated with temporary Naso phases [35]. In Sweden, a temporary Naso trial has historically been used to avoid unnecessary PEG‑J surgery, reflecting the national development of both LCIG and LECIG [36]. Given the limited evidence supporting mandatory Naso testing [19, 35] and findings from the present study further question its value given the distress it caused participants. Clear communication about its temporary nature and timely support appear important for perseverance. For centres where Naso trials remain part of routine practice, structured preparation for both PwPD and FC may help reduce anxiety and improve treatment readiness. These findings also support ongoing discussions regarding whether routine Naso testing should remain part of contemporary DAT pathways or be reserved for selected cases.

Studies on LCIG and CSAI similarly report that while PwPD often adapt well and regain time for valued activities, caregivers continue to face technical and organisational demands beyond the initiation phase [36–39]. Our findings suggest this complexity: FC valued increased stability but described emotional and logistical burdens that persisted over time. Research on DBS has characterized a four-phase caregiver adaptation trajectory: decision-making, initiation, adjustment, and re-adaptation [40]. While our study did not explicitly explore these phases, the ongoing practical and emotional challenges reported by FC suggest that comparable transitions may occur with pump therapy. Importantly, caregiver involvement varied across patient-caregiver dyads. While some FC described providing only limited practical assistance, others reported substantial responsibility for practical management and troubleshooting. This variation suggests that caregiver burden is influenced not only by the therapy itself but also by how responsibilities are distributed within the dyad.

### Finding 2: Sustaining hope in the face of therapeutic uncertainty

Consistent with this finding, overall satisfaction depended on multiple factors, including the practical demands of managing a complex device. Echoing DBS research, participants described an initial phase of high expectations followed by a more reconciled acceptance of residual symptoms. This illustrates the dynamic nature of expectation adjustment [41]. In our study, informational gaps, particularly regarding practical workloads and adjustment periods, contributed to uncertainty and occasional disappointment. These findings align with previous research documenting unmet informational needs in advanced PD care [42, 43]. Preparing patients and caregivers with clear guidance and emotional support is therefore essential for aligning expectations with lived experience and fostering sustained engagement with DAT [13, 23, 44].

### Finding 3: Relying on expertise while managing its absence

Criticism of poor equipment design reflected a broader perception that DAT requires continuous effort rather than seamless integration. Addressing these challenges requires sustained practical and emotional support and attention to device usability and caregiver training, echoing earlier calls for structured interventions to reduce technical burden and caregiver strain [21, 45]. Specialist PD nurses play a pivotal role in facilitating successful DAT adaptation, through reassurance and practical guidance in early phases [42, 45]. When this support is lacking, care gaps emerge, shifting technical and educational responsibilities to families [21, 37, 46]. Participants described how specialist support was readily available during treatment initiation but became less accessible over time, forcing many to rely on manuals and personal initiative. Recent evidence from Denmark suggests that home-based LCIG titration is both feasible and well accepted, providing an alternative model of care that may reduce some of the logistical burdens associated with treatment initiation while increasing access to specialist support [47]. Such approaches may represent one way of addressing the continuity gaps identified in the present study. Similar gaps have been documented internationally, underscoring the need for system-level strategies to ensure continuity of care in light of a progressive disease [48].

Collectively, these findings highlight the need for multidisciplinary support that extends beyond treatment initiation and includes structured education for both FC and general HCP [46, 49]. Although multidisciplinary decision-making is recommended in national guidelines [6] long-term practical and emotional support remains insufficiently integrated into routine care. The support needs described by participants extended beyond treatment initiation and technical training. Taken together, these findings suggest a need for more sustainable care models that integrate person-centred and palliative principles throughout the DAT trajectory [48–50]. Rather than focusing solely on treatment initiation and symptom control, support should address the evolving informational, practical, and emotional needs of both PwPD and FC.

### Overall interpretation

Taken together, our findings illustrate that adaptation to pump therapy is not a “set-and-forget” process, but a relational and contextual journey shaped by expectations, practical challenges, and evolving support needs. Our findings reinforce the notion that adaptation to advanced therapy is shared rather than individual, consistent with previous work emphasizing the importance of “being in it together” during treatment transitions [51].

### Strenghts and limitation

While the study design allowed participants to reflect across the trajectory of therapy, it also introduced limitations. Recall accuracy may have been affected, particularly among PwPD with cognitive symptoms, who at times struggled to distinguish between the Naso trial and PEG phases. Given that interviews were conducted 6–24 months after treatment initiation, some participants may have merged experiences from different stages of the treatment trajectory or assigned specific challenges to the wrong phase. This may have influenced the precision of accounts related to treatment initiation, although broader reflections on adaptation, expectations, and support appeared consistent across participants. Furthermore, the sample consisted only of individuals who proceeded with pump therapy. Perspectives from those who declined DAT or discontinued treatment were not captured. Although participants described persistent symptoms, adverse effects, and treatment-related challenges, all interviewed PwPD continued pump therapy at the time of the study. There is also a risk that both highly burdened and particularly well‑functioning individuals are underrepresented, as non‑participants most often declined due to health issues or time constraints. One PwPD participated without a FC, but the account did not differ substantially from those of other participants. Employment status among FC was not considered and may have influenced perceived burden. Consequently, transferability of findings, particularly regarding decision-making processes and barriers to treatment adaptation, may be limited.

A major strength of the study is the inclusion of both PwPD and FC perspectives. Previous qualitative studies have largely focused on patients, whereas the present study enabled exploration of how adaptation to pump therapy is experienced and negotiated within an ongoing care relationship. Inclusion of participants from two geographically distinct centres further strengthened transferability by capturing experiences across different organisational contexts. This design provided novel insights into the relational, practical, and emotional aspects of adaptation to levodopa-based pump therapy.

## Conclusions

This study demonstrates that expectations, lived experiences of pump therapy and professional support are closely intertwined in shaping adaptation to DAT. Including both PwPD and FC perspectives reveals how decisions and adjustment processes unfold within an ongoing care relationship. Although participants reported improved symptom control and daily routines, adaptation was not a “set-and-forget” process but a relational and contextual journey requiring ongoing informational, emotional, and practical support. These findings emphasize the need for coordinated, person-centred care that supports both PwPD and FC and ensures continuity across the treatment trajectory. Future research should examine why some individuals decline DAT and evaluate the impact of structured preparation and follow-up programmes on long-term satisfaction and caregiver burden. 

## Supplementary Information

Below is the link to the electronic supplementary material.

Supplementary Material 1: Semi-structured Interview Guides (PwPD and FC).

Supplementary Material 2: Representative quotations per theme/subtheme.

## Data Availability

The dataset contains sensitive information and is not publicly shared to protect participant anonymity and confidentiality. Anonymized data may be made available from the corresponding author upon reasonable request, provided that such sharing complies with ethical and legal requirements.

## Abbreviations

CSAI: Continuous Subcutaneous Apomorphine Infusion
DAT: Device-aided Therapy
DBS: Deep Brain Stimulation
FC: Family Caregiver
HCP: Health Care Professional
LCIG: Levodopa/Carbidopa Intestinal Gel
LDp/CDp: foslevodopa/foscarbidopa
LECIG: Lecigon/entacapone/Carbidopa Intestinal Gel
PD: Parkinson Disease
PEG: Percutaneous Endoscopic Gastrostomy
PwPD: Person with Parkinson Disease
