# Supplementary material for: Initiating levodopa pump therapy: a qualitative study of patient and caregiver perspectives
Source: BMC Nurs. 2026 Oct 20;25:1001. doi: 10.1186/s12912-026-05527-6 (PMC13689137; doi:10.1186/s12912-026-05527-6)
Supplement: Supplementary file 1 [file 12912_2026_5527_MOESM1_ESM.docx]

**Additional file 1: Semi-structured Interview Guides (PwPD and FC).**

***Interview Guide – Patient***

| *My name is…*  *We are interested in your reflections, opinions, and thoughts.*  *How the information will be handled (audio recording, transcription, pseudo-anonymization).*  *Do you have any questions, and can we have your verbal consent to continue?* | |
| --- | --- |
| ***Theme: Life with Parkinson’s disease during a complicated phase*** | |
| *Question* | *Probes* |
| **Can you describe what life was like for you shortly before you were offered pump therapy?** | *How did it affect your daily life, social activities, and independence?*  *How did you feel about the future and the possibility of new treatments?* |
| ***Theme: The process of getting advanced treatment*** | |
| *Question* | *Probes* |
| **Can you describe the journey from hearing about pump treatment to deciding to accept it?** | *What information did you receive,*  *from whom,*  *and how did it help you make the decision?*  *Did you have any reservations or specific concerns?*  *How did your family or social network influence the decision?* |
| **Can you describe your thoughts and expectations about the pump treatment?** | *Which symptoms or aspects of life did you hope would improve?*  *Was there anything you were unsure about or afraid of losing with the treatment?* |
| ***Theme: Starting the treatment*** | |
| *Question* | *Probes* |
| **Can you tell me about your experiences when you started the treatment?** | *How did you experience the support from the care team?*  *Were there any positive or negative experiences at the beginning, for example during the initiation period?*  *Any feelings of euphoria?* |
| ***Theme: Life with pump treatment*** | |
| *Question* | *Probes* |
| **Has life with the pump turned out as you expected?** | *Advantages and challenges of the treatment?*  *How has it affected your daily life and well-being?*  *Has it changed your self-image or sense of independence?* |
| **If you met someone who is considering pump treatment, what would you tell them?** | *What do you think is important for them to know to prepare for what the treatment involves?* |
| **Is there anything else you think is important for me to know about your experience with pump treatment?** |  |

***Interview Guide – Relatives***

| *My name is…*  *We are interested in your reflections, opinions, and thoughts.*  *How the information will be handled (audio recording, transcription, pseudo-anonymization).*  *Do you have any questions, and can we have your verbal consent to continue?* | |
| --- | --- |
| ***Theme: Life with Parkinson’s disease during a complicated phase*** | |
| *Question* | *Probes* |
| **Can you describe what life was like for you shortly before your relative was offered pump therapy?** | *How did it affect your daily life, social activities, and independence?*  *How did you feel about the future and the possibility of new treatments?* |
| ***Theme: The process of getting advanced treatment*** | |
| *Question* | *Probes* |
| **Can you describe the journey from hearing about pump treatment to your relative deciding to accept it?** | *What information did you receive, from whom, and how did it help you make the decision?*  *Did you have any reservations or specific concerns?*  *How did your family or social network influence the decision?* |
| **Can you describe your thoughts and expectations about the pump treatment?**  **What did you think it would mean for you?** | *Which symptoms or aspects of life did you hope would improve?*  *Was there anything you were unsure about or afraid of losing with the treatment?* |
| ***Theme: Starting the treatment*** | |
| *Question* | *Probes* |
| **Can you tell me about your experiences when your relative started the treatment?** | *How did you experience the support from the care team?*  *Were there any positive or negative experiences at the beginning, for example during the initiation period?*  *Any feelings of euphoria?* |
| ***Theme: Life with pump treatment*** | |
| Question | Probes |
| **Has life with the pump turned out as you expected?** | *Advantages and challenges of the treatment?*  *How has it affected your daily life and well-being?* |
| **If you met someone who is considering pump treatment, what would you tell them?** | *What do you think is important for them to know to prepare for what the treatment involves?* |
| **Is there anything else you think is important for me to know about your experience with pump treatment?** |  |
