# Supplementary material for: Initiating levodopa pump therapy: a qualitative study of patient and caregiver perspectives
Source: BMC Nurs. 2026 Oct 20;25:1001. doi: 10.1186/s12912-026-05527-6 (PMC13689137; doi:10.1186/s12912-026-05527-6)
Supplement: Supplementary file 2 [file 12912_2026_5527_MOESM2_ESM.docx]

**Additional file 2: Representative quotations per theme/subtheme.**

| **Theme/subtheme** | **Quotes** |
| --- | --- |
| Living with the constraints of PD  Symptoms of PD  Cycle of loss  Pills, pills, pills!  Shrinking world  Emotional implications | “But suddenly, it hits, and that’s what I think is so unpredictable about the disease…//That’s what bothers me, that you go in feeling fine and come out sick.” (PwPD3)  “I was playing mini golf at [club name] and working there, and I often delayed taking my pills. Then suddenly, I’d feel unwell and had to take extra medication to recover. It was a constant struggle. If I missed a dose, I’d go into an ‘off’ state.” (PwPD4)  “Yeah... Pills, pills, pills! Well, it wasn’t that many, really, but... I had never in my whole life taken any pills, so it was new to me.” (PwPD8)  “No, but because it was, you know, a tablet every 90 minutes. And that, and I mean, it’s not easy to remember, right? No, because it was a constant thinking about time, I would say. Because I also knew that if they forgot about it—because, my God, it’s not easy for them to remember either—no, and… and I mean, if they forgot about it, you know, then they would feel bad, yeah.” (FC4)  “It wasn’t just difficult to plan from day to day but even within the same day. It went up and down all the time. //…// I couldn’t decide anything on my own either because they might need my help to get somewhere.” (FC7)  “When it was with the tablets, I kind of felt a bit guilty, like, why didn’t I… Maybe I didn’t remind them or something, you know?” (FC8) |
| Sustaining hope in the face of therapeutic uncertainty  Information and communication about DAT  Feeling cornered  Expectations about the pump  Satisfaction with the pump therapy | “I trust the healthcare system, and they wouldn’t have given it to us or said anything if it couldn’t help us.” (FC 1)  “Yes... Of course, you think, you think: now I have to have this. In any case, I wouldn’t have gotten this…[without help from FC8] //…// Otherwise, well... you don’t really have many options either, it’s either... I take lots of pills or I have something like this [the pump].” (PwPD8)  “But it sounded good. And I... was eager to try. Yes, out of curiosity, because you can always go back to pills if it didn’t work. You have to keep a door open to go back.” (PwPD9)  “We had great expectation. We thought that the therapy would be able to turn back time a couple of years.” (FC5)  “Yes, exactly, it’s really difficult to get a clear idea of how and what it actually involves. It was more about the appearance.” (FC5)  “And we have to admit that it’s one thing to look at the equipment [the pump], but it’s a whole other thing to actually handle it.” (FC5) |
| Adapting to a new treatment while facing practical challenges  Effect of the pump  Adjusting and managing the pump  PD is progression  PwPD: Starting with Naso tube  PwPD: Impact on social relations  FC: Inserting Naso/PEG | “But one thing I've also done is forced myself to go out. And have guests and so on. Often and such. But eventually, I have to force myself. Because we could talk, and it wasn't fun. Yeah, but we’re going to our grandchildren. No, it's not fun. But I went anyway. And now it's different. Yeah. Since I got the pump.” (PwPD 7)  “No, it's so stiff! So that's the only thing, otherwise, I could have done it. It's really hard to get in!” (PwPD1)  “[regarding the carring material] Yes, what comes with the pump is useless... I think it’s very strange that they don’t have a better option. Yeah, anything is better than the one that comes with it [the pump]” (FC7)  "And so… My memory has changed too, I notice, very much. [crying tone]" (PwPD2)  “Then it just got worse. The doctor said it would settle down and go away. But she was shaking so much that she fell off the couch.” (FC1)  “But I got a really sore throat from that tube, the one through my nose, and trying that… That was when I almost backed out. But they explained to me that it wouldn’t be the same problem at all once I… once I got it properly in my stomach. Because, you know, it’s not easy when it’s just hanging down into your throat—it’s all mixed in with food, air, everything. [clears throat]. I got really bad acid reflux from it.” (PwPD6)  Just that I can take part in everything, we can go to IKEA or something [laughs]. But like, I can be there, and in the beginning, we would borrow a wheelchair if we were going to walk around for a long time. And it’s just that I can be there, and we had coffee there, and yeah, we can do other things. You can do a bit more.” PwPD1  “I think it was more for them than we expected. They found it significantly more difficult to go through with it. That’s how it was, really. And the time spent in the hospital, up in neurology, when they were there—it was quite tough for them, from what I experienced. I think it was a bit more than we had anticipated.” (FC5) |
| Relying on expertise while managing its absence  HCP knowledge about pump  Support from PD/pump support nurses  FC: FC support, receiving and giving | “No, I mean, no one really knows what to do with this pump. No one knows how it works. They [general HCP] don’t even know what it is.” (PwPD7)  “Yes, the support has been really good. That's how it is. Lucky that it exists. So that it's easy to look up and call them. And there has been trouble, so talk to them so that it...” (PwPD 9)  “So far, I’ve gone shopping once a week, and I go to the gym, which is just across the river—a short trip, about an hour. I say, "Now don’t do anything silly while I’m gone." I say it jokingly, but still... It’s a bit of a concern.” (FC2) |
